# Influence of Androgens on Immunity to Self and Foreign: Effects on Immunity and Cancer

**DOI:** 10.3389/fimmu.2020.01184

**Published:** 2020-07-02

**Authors:** Isabel Ben-Batalla, María Elena Vargas-Delgado, Gunhild von Amsberg, Melanie Janning, Sonja Loges

**Affiliations:** ^1^Department of Oncology, Hematology and Bone Marrow Transplantation with Section Pneumology, Hubertus Wald Comprehensive Cancer Center Hamburg, University Medical Center Hamburg-Eppendorf, Hamburg, Germany; ^2^Department of Tumor Biology, Center of Experimental Medicine, University Medical Center Hamburg-Eppendorf, Hamburg, Germany; ^3^Martini-Clinic, Prostate Cancer Center, University Medical Center Hamburg-Eppendorf, Hamburg, Germany; ^4^Division of Personalized Medical Oncology, German Cancer Research Center (DKFZ), Heidelberg, Germany; ^5^Department of Personalized Oncology, University Hospital Mannheim, Mannheim, Germany

**Keywords:** androgens, immunity, cancer, immune cells, immunotherapy

## Abstract

It is well-known that sex hormones can directly and indirectly influence immune cell function. Different studies support a suppressive role of androgens on different components of the immune system by decreasing antibody production, T cell proliferation, NK cytotoxicity, and stimulating the production of anti-inflammatory cytokines. Androgen receptors have also been detected in many different cells of hematopoietic origin leading to direct effects of their ligands on the development and function of the immune system. The immunosuppressive properties of androgens could contribute to gender dimorphisms in autoimmune and infectious disease and thereby also hamper immune surveillance of tumors. Consistently, females generally are more prone to autoimmunity, while relatively less susceptible to infections, and have lower incidence and mortality of the majority of cancers compared to males. Some studies show that androgen deprivation therapy (ADT) can induce expansion of naïve T cells and increase T-cell responses. Emerging clinical data also reveal that ADT might enhance the efficacy of various immunotherapies including immune checkpoint blockade. In this review, we will discuss the potential role of androgens and their receptors in the immune responses in the context of different diseases. A particular focus will be on cancer, highlighting the effect of androgens on immune surveillance, tumor biology and on the efficacy of anti-cancer therapies including emerging immune therapies.

## Introduction

It has been known for a long time that sex is a biological variable directly affecting the immune response. Females are able to elicit stronger immune responses compared to males, leading to increased susceptibility to autoimmune diseases, while being less prone to infectious and malignant diseases (1). Sexual dimorphism in immunity has been attributed to a number of different factors, both endogenous, and environmental. Amongst the endogenous factors, one of the main contributors are sex hormones: estrogens and androgens (2). Androgens represent the male sex hormones, whose principal role is to trigger the development of male characteristics. They exert their biological functions through binding and activating the androgen receptor (AR) (3). Several studies have shown that androgens/AR are involved in immunomodulation, thereby impacting innate and adaptive immunity. Altogether, they have been shown to induce different immunosuppressive effects: decreasing antibody production levels, lowering T cell numbers and activation capacity, and stimulating anti-inflammatory cytokine production by antigen presenting cells (4).

Many cancers entities are affected by activation of the androgen/AR signaling axis, resulting in more aggressive phenotypes which can be in some cases inhibited by androgen deprivation treatment (please see below) (5). Moreover, cancer is ultimately the result of failed immune surveillance. In this respect, immunosuppressive effects of androgens could dampen anticancer immunity and contribute to the male predominance apparent in most cancers (6).

In this review, we will discuss how androgens and the AR influence immune cells and cancer incidence and progression. Finally, we will discuss what is known about the impact of male sex and androgens on the efficacy of different immune therapies in mice and humans.

## Androgens

Steroid hormones are a group of cholesterol-derived hormones. They are produced by different tissues including the adrenal cortex, testes, ovaries, adipose tissue, breast, endometrium, prostate, skin, salivary gland, kidney, and by the placenta during pregnancy (7). Based on their receptors, steroid hormones are classified into five groups: glucocorticoids, mineralocorticoids, androgens, estrogens, and progestogens.

The term “androgen” refers to any steroid hormone that has masculinizing effects (8). The biological actions of androgens, including testosterone and dihydrotestosterone (DHT) as well as androstenedione, dehydroepiandrosterone (DHEA) and its sulfated form (DHEA-S), are normally mediated through the androgen receptor (AR), a ligand-dependent nuclear transcription factor (9). After androgens are synthesized they are secreted into the blood stream predominantly as testosterone, which is mostly bound to sex hormone-binding globulin (SHBG). A very small fraction of testosterone (<3%) circulates as a free bioavailable form. Due to the high affinity of SHBG for testosterone, this globulin is regulating the amount of unbound testosterone available for target tissues (10). After entering its target cells, testosterone is converted to the most biological active form of androgens, dihydrotestosterone (DHT) by the enzyme 5α-reductase in most of the male reproductive organs. Testosterone can be also metabolized by aromatase into estradiol, primarily in fat tissues and in the hypothalamus, as well as in hematopoietic cells (11, 12). Therefore, local sex-hormone-mediated effects will be determined by the expression levels of either enzyme, as this will directly regulate the balance between androgen and estrogen production (13). This needs to be taken into account also in the context of androgen-mediated effects on immune cells (please see below). Careful experimental design is warranted in order to proof that observed phenotypes are directly caused by testosterone. Androgens, mainly testosterone and DHT, are the male sex hormones required for development of the male reproductive system and secondary sexual characteristics. In physiological conditions testosterone stimulates not only psychosexual behavior, but also physical and functional features. They include spermatogenesis, formation of the Wolffian duct, development of a deeper voice, bone mass, musculature, axillary, and pubic hair (3). DHT is responsible for the growth of the prostate and the external genitalia, as well as for male pattern of hair growth on the face and body and for male androgenic alopecia (12, 14). In summary, testosterone is more important in mediating anabolic effects while DHT is more potent in exerting androgenic effects.

## Androgen Receptor Mediates Androgen Effects

### Androgen Receptor Signaling

Most biological actions of androgens are mediated via the nuclear androgen receptor (AR). AR is a ligand-dependent nuclear transcription factor that belongs to the steroid hormone nuclear receptor family together with other members, including the estrogen receptor (ER), glucocorticoid receptor (GR), progesterone receptor (PR), and mineralocorticoid receptor (MR) (15).

There are two different pathways of androgen signaling, the canonical or genomic and the non-genomic or non-classical pathway (16, 17). Signal transduction through the classical AR happens in several steps. In the absence of androgens, AR is located exclusively in the cytoplasm and associated with heat-shock proteins (HSPs). Binding to the ligand induces the dissociation of AR and HSPs and leads the subsequent translocation of AR to the nucleus (18). Once AR is shuttled into the nucleus, ligand-activated AR binds specific DNA regulatory sequences [androgen response elements (ARES)] (19). This ligand-dependent transcription factor modulates gene expression through direct DNA binding and the recruitment of several coregulators to form complexes, which are necessary to induce epigenetic histone modifications and chromatin remodeling at target genetic loci (20, 21).

The activation of the non-genomic or non-classical pathway leads to rapid, transcription-independent effects of androgens caused by their binding to non-classical receptors including ZIP9 and GPRC6A (16, 17), which affect the regulation of other transcription factors, nuclear receptors and cytoplasmic signaling events. Non-classical receptors can also be associated to G-proteins in the plasma membrane (22, 23). Examples of effects induced by binding of androgens to non-classical receptors include activation of mitogen-activated kinase (MAPK), protein kinase C (PKC), protein kinase A (PKA), and increases in free intracellular calcium. In addition, AR can also be transactivated in presence of very low levels or absence of DHT via different cell surface receptors such as HER2. Signal activation emerges from different mechanisms, which are not necessarily mutually exclusive, including extracellular signaling peptides such as interleukin-6 (IL-6), epidermal growth factor (EGF), and insulin-like growth factor (IFG). Altogether, the androgen-independent activation of AR occurs relatively often in cancer (22, 24).

### The Role of Androgen Receptor in Health

In physiological conditions, the main role of the androgens/AR axis is the development of male characteristics, including spermatogenesis and the mediation of neurobiological and behavioral sex differences between female and male mammals already during the perinatal development (25). Behavioral gender dimorphism can be reflected in aggressiveness, parental care, or territorial behavior for example. Not only testosterone is necessary, but also estrogens are required during the early neonatal period for the development of male behavior traits. Here, testosterone is converted in estrogens through aromatase activity. This enzyme is expressed in brain cells including neurons. Interestingly, neurons expressing aromatase showed sex difference in their location within the brain. It was found that male mice had higher numbers of aromatase-positive neurons in the areas of the brain responsible for modulating aggressive and sexual behavior. In addition, it was demonstrated that estrogens are capable to masculinize aromatase positive neurons in these regions, contributing to the development of male behavior (26). In line with this, similar effects were observed in humans diagnosed with psychiatric syndromes, where males showed more aggressive behavior (27).

The AR is expressed in a diverse range of tissues and systems, besides the male reproductive organs. It can be found in muscle, bone, and adipose tissue, as well as in the immune, cardiovascular, neural, and hematopoietic systems, in which androgens have also been documented to exert biological actions (15, 28).

The bone represents the most important extragonadal site influenced by androgens/AR. In this regard, testosterone has important effects on bone physiology because when men are hypogonadal, a condition with too low levels of this hormone, bone homeostasis is severely perturbed. This perturbation results in osteopenia of regions richer in cortical bone, such as the radius, and in trabecular bone like the spine. These effects can be reversed upon replacement with testosterone (29). The effect of testosterone treatment on the bone of women with low serum levels of this hormone is not clear but probably small.

Another relevant extragonadal site of androgen influence is the cardiovascular system. Cardiovascular diseases are known to have significant sex disparity, with men presenting earlier onset and greater severity compared to women. Specifically, men have a 2 to 3-fold higher age-specific risk of cardiovascular death (30). Preclinical studies in a mouse model of pressure overload by transaortic constriction induced cardiac hypertrophy showed that treatment with a DHT conversion inhibitor, finasteride, reduced mortality in both sexes diminishing ventricular dilation and dysfunction, as well as pathological cardiac hypertrophy and fibrosis (31). Similarly, after orchiectomy in mice, detrimental cardiac remodeling and dysfunction generated by several stressors, was prevented with the removal of androgens (32). Altogether, increased androgen levels are important for cardiac pathophysiology.

### Effects Androgen Receptor Mutations

Loss-of-function mutations are key players in the modulation of receptor functions. They can lead to changes in the structure of an encoded protein resulting in a decrease or complete loss of its expression. AR is located on the X chromosome and mutations are relatively common. In this context, androgen insensitivity is the most frequent form of genetic hormone resistance. Since males carry one copy of X chromosome, AR mutations with functional consequences are definitely expressed in all cells of affected males. In contrast, females bearing these mutations are silent carriers without any obvious phenotype because the functional allele on their second X chromosome will mostly counteract the effect. Nevertheless, there are some exceptions in which a small percentage of women (~10%) carrying AR mutations exhibit mild phenotypic effects including mildly decreased body hair, delayed puberty onset, and/or increased height (33, 34). Due to the overall suppressive effect of androgens on lymphocytes (please see below), it would be very interesting to find out whether lymphocytes with an AR-mutated allele would be positively selected over cells carrying WT AR allele, as this is currently unknown. Further research is necessary to answer this relevant question.

Regarding the AR gene, many different types of mutations have been described. The most common comprise perturbation of the reading frame caused by insertions, deletions, splice site interruptions, and frame-shifts which often compromise protein function. Moreover, another typical mutation is single base replacement, whose effects can differ from no effect to a complete loss-of-function. In addition, other less frequent inactivation mechanisms induced by mutations exist such as for example loss of conformational stability resulting in inefficient or aberrant translation thereby diminishing the expression of functional AR protein.

Mutations of which the vast majority (more than 90%) are single base replacements occur at multiple loci within the AR gene. They have been shown to result in pathophysiological consequences when amino acid substitution takes place in the functionally crucial regions including the DNA-binding domain (DBD) or ligand-binding domain (LBD). However, if mutations occur in non-functional regions they can also represent silent polymorphisms (35). When AR mutations happen in the germline, the situation is similar, a broad spectrum of functional consequences can result ranging from absence of phenotypic changes to different androgen insensitivity syndromes (AIS). AIS exhibit different phenotypes correlated with the degree of impairment of AR function. The clinical consequences can be classified as complete, partial or mild. Complete androgen insensitivity syndrome in men (CAIS, previously known as testicular feminization) results in an undervirilized male phenotype with impaired differentiation of male gonadal tissue and incomplete development of the external or internal genitalia. Nevertheless, seemingly normal male phenotypes can also occur. Partial androgen insensitivity syndrome (PAIS) is characterized by impaired male genitalia development, showing external genital feminization and secondary sexual characteristics like breast development. The degree of conversion from male to female phenotype is correlated with the severity in which the mutation functionally affects the AR. Mild androgen insensitivity (MAIS) is the least severe form of androgen insensitivity. It can be sufficient to minimally impair spermatogenesis, but these individuals display normal male genital differentiation, with only discreet changes in body habitus and size, as well as in face and body hair patterns (33, 36).

To corroborate the key role of AR mediating the biological effects of androgens genetic mouse models have been generated, in which the gene encoding AR has been knocked out (37–41). A phenotypic analysis performed in ARKO male mice showed that they have a female-like appearance and reduced body weight, compared to male wild-type (WT) mice. They have also about 80% smaller testes and lower concentrations of serum testosterone. Additional features include incomplete spermatogenesis, increased number and size of adipocytes, as well as reduced cancellous bone volumes compared with WT littermates. Moreover, in female ARKO mice, the average number of pups per litter is lower than in WT female mice, regardless of homo- or heterozygous genotype, pointing to possible defects in female ovulation and fertility (41).

Altogether, androgens and AR exert a central role in health and pathophysiology. Therefore, it is important to dissect the biological effects of this axis in different contexts including the immune system.

## Effects of Androgens on the Immune System

Beyond the roles described above for androgens/AR in regulating the male phenotype development, it has been demonstrated that they can also regulate immune function. AR can act directly on immune cells by influencing the transcription of immune-regulatory genes through DNA-binding-dependent and -independent mechanisms (21). Immune modulation exerted by androgens has been investigated in animal models and humans. These studies put forward androgens as important drivers of the well-described gender dimorphism in infectious and autoimmune diseases, with females being usually more susceptible to autoimmunity diseases, and less vulnerable to infections than males (42). In this context, it has to be taken into account that sex differences in immunity cannot be attributed solely to sex hormones but are multifactorial in origin and include effects due to X-chromosome inactivation and behavioral differences amongst others. It is beyond the scope of this review which is focused on androgens to discuss all factors potentially influencing the sex bias of the immune system and we refer the reader to recent comprehensive reviews in this field (1, 2, 43).

It was found that AR are expressed in a wide variety of innate and adaptive immune cells including neutrophils, macrophages, mast cells, monocytes, megakaryocytes, B cells, and T cells (44–52). Interestingly, AR are expressed also in hematopoietic stem cells and lymphoid and myeloid progenitor cells (44, 53, 54). For a comprehensive overview table of the different hematopoietic cell populations and their AR expression we refer the reader to a comprehensive review covering this topic (44). Therefore, androgens can directly influence both the progenitor and mature immune cell compartment.

**Figure 1:**
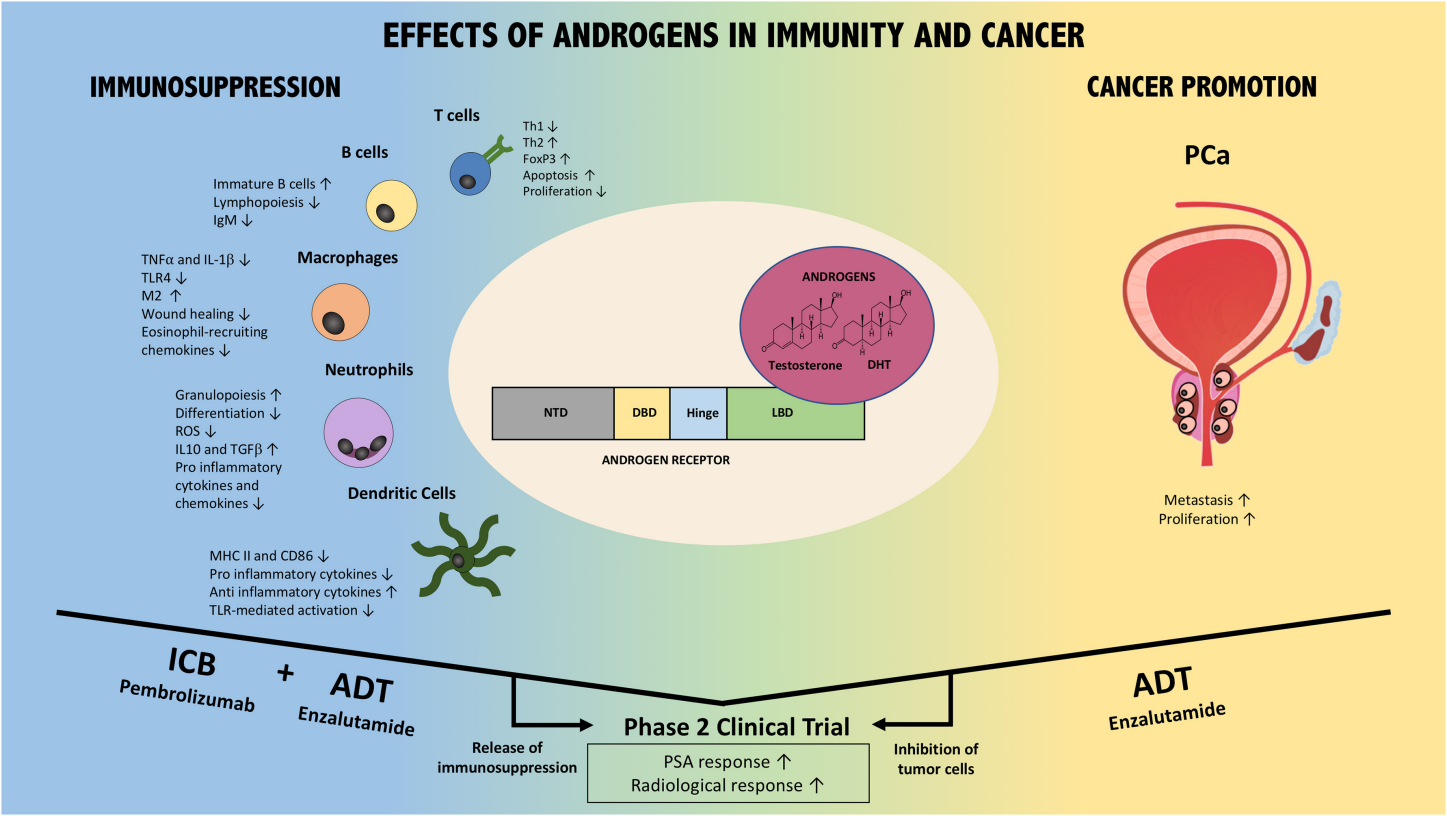
Effects of AR/Androgen signaling in immunity and prostate cancer. AR/androgens can influence different immune cell subsets, including T cells, B cells, macrophages, neutrophils, and dendritic cells (Left part of the figure). Overall, their effect is immunosuppressive. In addition, androgens/AR directly and indirectly promote prostate cancer (PCa) via different mechanisms (Right part of the figure). Thus, the combination of ADT with immune checkpoint blockade could foster anti-tumor immune responses (ICB+ADT) while ADT additionally inhibits PCa directly. This combination strategy has resulted in improved patient responses compared to either monotherapy in Phase 2 clinical trials. Confirmatory Phase 3 trials are warranted and ongoing. NTD, N-terminal domain; DBD, DNA binding domain; LBD, ligand biding domain; DHT, dihydrotestosterone; ADT, androgen deprivation therapy; ICB, immune checkpoint blockade; PCa, prostate cancer; PSA, prostate specific antigen.

Evidence derived from different studies points to a rather immunosuppressive role of androgens in different immune cell types mostly by reducing and/or promoting expression of pro-inflammatory and anti-inflammatory mediators, respectively [Figure 1; (55)]. In the following section, we will discuss what is known about the effects of androgens and AR in different innate and adaptive immune cells.

### Neutrophils

Neutrophils, also coined polymorphonuclear (PMN) leukocytes, are the major cell type in human blood, and are considered the first line of defense in the innate immune system response. Their function is to identify and attack invasive microorganisms through phagocytosis and degrade the pathogens intracellularly. As consequence, granular material is released and neutrophils extracellular traps (NETs) are generated, helping to kill more pathogens (56).

AR is expressed in the majority of neutrophil lineages, including proliferative precursors like promyelocytes, myelocytes, and myeloblasts, as well as in mature neutrophils. AR expression patterns were not found to be differentially affected by gender in these cells (45). Androgens can promote neutrophil differentiation and recruitment, thereby increasing their numbers in mice and humans (44, 57, 58). Consistently, neutrophil numbers are decreased after castration and in ARKO- and Tfm mice, indicating that androgen and AR signals positively regulate neutrophil development. For example, ARKO mice have severe neutropenia with only one-tenth of the neutrophils of WT mice. Further analyses of the neutrophil lineage in ARKO mice showed that precursors and mature neutrophils are significantly reduced (59). In line with these findings, prostate cancer patients with drug-induced androgen blockade also display neutropenia (60, 61). Moreover, in addition to reduced neutrophil counts, functional defects of neutrophils were also observed in ARKO mice: neutrophils retain normal phagocytosis properties but respond less to granulocyte-colony stimulating factor-induced proliferation and to migratory signals *in vitro*. In addition, they are more susceptible to apoptosis and produce less proinflammatory cytokines (IL-1β, IL-6, and TNF-α) and chemokines (CCL2, CCL3, CCL4, CXCL1, CXCL4, and CXCL7) compared to neutrophils from WT mice (62). Altogether, these findings show that androgens/AR are important for neutrophil development and some important aspects of their functionality.

On the other hand, it was observed that testosterone can also foster the maintenance of immunosuppressive neutrophils pointing toward a novel mechanism of protection against autoimmune disease including the development of lupus-like disease in lupus-prone (NZB × NZW)F1 male mice. Here, Gr-1^high^Ly-6G^+^CD11b^+^ myeloid-derived suppressor cells (MDSCs), a heterogeneous population of immature myeloid cells, displaying a neutrophilic nuclear morphology and immunoinhibitory action, were constitutively increased in male BWF1 mice compared to female mice, which was regulated by testosterone (63).

Furthermore, by using a bacterial model of prostate inflammation in male rats, it was shown that testosterone induces impaired myeloperoxidase and bactericidal activity in neutrophils. In addition to reduced functionality, an increase in the expression of the anti-inflammatory cytokines IL-10 and TGFβ1 was also observed, similar to what is observed in immunosuppressive “N2-like” neutrophils, which reside within the tumor microenvironment. These data reveal an interesting function of testosterone promoting inefficient and anti-inflammatory neutrophils leading to prolonged bacterial inflammation and an appropriate environment for several infectious diseases (57).

In summary, available literature indicates a dual and partly contradictory role of androgens/AR with a positive effect on neutrophil differentiation and some facets of their pro-inflammatory role while they can also support an immunosuppressive phenotype and inhibit bactericidal properties. Thus, more functional research and importantly investigations in humans are warranted to dissect the impact of androgens on neutrophils in health and disease.

### Macrophages

Macrophages and monocytes, their precursors, are the “big eaters” of the immune system. They represent specialized cells involved in the detection, phagocytosis and destruction of bacteria and other foreign and harmful microorganisms. Macrophages are present in every tissue of the body. They originate from monocytes, which are quickly recruited upon infection or tissue damage, leading to their differentiation into tissue-specific macrophages. Their main function is to engulf pathogens or apoptotic cells and generate immune effector molecules. Moreover, macrophages are antigen presenting cells (APCs) that interact with T cells and initiate inflammatory response by releasing cytokines activating different populations of immune cells (64). Altogether, they play an important role in atherosclerosis, infections and wound healing. Both monocytes and macrophages were found to express AR, which was confirmed by functional studies (45, 47, 65).

The impact of androgens on macrophage function has been addressed in several studies and overall point to an (immuno)suppressive effect. For example, it was found that castrated male mice are significantly more susceptible to endotoxic shock, which results from a severe and generalized pro-inflammatory response induced by systemic infection with gram-negative bacteria. Notably, this effect was reverted when the mice were treated with exogenous testosterone (66).

In line with this, a complex network of cytokine and immune cell interactions is present during sepsis in humans leading to high mortality due to organ dysfunction or failure. Macrophages are recognized to play essential roles in sepsis and influence both inflammatory responses and immune homeostasis. Besides, macrophage dysfunction is recognized as one of the main causes for sepsis-induced immunosuppression in mice and humans (67). Epidemiological studies identified male gender as an independent risk factor for the development of severe infection compared with females. Male sex hormones have been shown to have a suppressive effect on cell-mediated immune responses, including in macrophages. Consistent with this notion, it has been demonstrated in humans, that the female sex is protected from septic conditions requiring an active cell-mediated immune response, whereas the male sex has been associated to suffer deleterious consequences due to a reduced cell-mediated immune response, where splenic and peritoneal macrophage cytokine release are depressed. Remarkably, in preclinical models, administration of the anti-androgen flutamide after the induction of sepsis, was able not only to reestablish the low cytokine released levels by splenic macrophages and splenocytes, but also significantly decreased the mortality of post-hemorrhaged mice (68).

Rettew et al. demonstrated that testosterone could reduce the expression of Toll-like receptor 4 (TLR4) in a macrophage cell-line, in cultured primary macrophages and *in vivo* in mice (66). TLR4 is a transmembrane receptor that when activated leads to intracellular NF-κB signaling pathway induction and inflammatory cytokine production, promoting the activation of the innate immune system (69). However, more research is warranted to demonstrate a direct effect of androgens on the function and phenotype of macrophages.

Chronic inflammation induced by macrophages is strongly associated with cardiovascular disease. Inflammation is a key player in the development and progression of coronary heart disease (CHD) and testosterone has been shown to dampen the inflammatory response by suppressing the expression of TNF-α and IL-1β in stimulated human macrophages cultured *in vitro*. These results need functional validation in an *in vivo* setting, but lead to the hypothesis that testosterone could exert an anti-inflammatory effect on macrophages which could be explored in the CHD setting (70).

An unexpected role for androgen/AR was found in promoting M2 polarization of alveolar macrophages (AM), which correlates with asthma severity in humans. Asthmatic women present more M2 macrophages than asthmatic men, therefore androgens were used as an experimental asthma treatment. Using mice lacking AR specifically in monocytes/macrophages (AR^flox^LysMCre), was observed only in males, and impaired M2 polarization leading to lung inflammation and reduced eosinophil recruitment, which could be due to a reduction in eosinophil-recruiting chemokines in alveolar macrophages deficient in AR (71).

On the other hand, castration of male mice or blockade of androgen action by flutamide hastened wound healing associated with lower macrophage infiltration, a dampened local inflammatory response and decreased expression of the proinflammatory cytokine TNF-α (72). This shows, that similar to the findings observed in neutrophils (please see above), androgens/AR mostly exert a negative influence on macrophage function, but can in certain conditions also promote their function.

### Dendritic Cells

Dendritic cells (DCs) are APCs derived from bone marrow precursors and are widely distributed across the body. DCs are a heterogeneous group capable of initiating and orchestrating immune responses, acting often as messengers between the innate and the adaptive immune system. Their main function is to process and present antigens via MHC molecules to T cells. DCs exert immune-surveillance for exogenous and endogenous antigens and induce the activation of naive T cells, thus, orchestrating diverse immunological responses (73).

Overall, testosterone induces an inhibitory effect on DCs, nevertheless it remains unclear whether it is a direct or indirect effect because the expression of AR by DCs has not been clearly determined (44). In this context, there is one study performed in mice showing that bone marrow-derived DC (BMDCs) express ER, but not AR (74). Conversely, another study indicates that production of anti-inflammatory cytokines by BMDCs was increased at low to medium DHT exposure, suggesting the presence of AR. Additionally, in the same study carried out in mice, ChIP analysis was performed with tumor associated DCs, as well as splenic DCs revealing ERα and AR expression by DCs from both tissues (75). In addition, ER expression was found in hepatic DCs, suggesting altogether an influence of sex hormones on DC function in mice (76). However, the evidence is scarce at this point, especially concerning direct effects of androgens on DCs and further research is warranted in order to dissect these effects and clarify the role of estrogens.

Viral infections lead to different clinical manifestations between sexes in humans, and it has been reported that this is also the case for HIV-1 disease development. One of the differences observed is that during the response to Toll-like receptor 7 (TLR7) ligands, which are encoded by HIV, the production of interferon-alpha (IFN-α) by female plasmacytoid DCs (pDCs) is significantly higher than the levels produced by male pDCs. Accordingly, women develop more robust secondary activation of CD8^+^ T cells. In line with these *in vitro* experiments, stronger CD8^+^ T cell activation in women chronically infected with HIV-1 was observed compared to men, after normalizing the viral load for all the patients. These results point out that sex differences observed in the progression of HIV-1 may be due to stronger immune responses in women, which present higher activation of pDCs induced by TLR compared to men at a given viral load (77).

Sex differences in DCs have also been demonstrated using a well-studied mouse model of infection with lymphocytic choriomeningitis virus (LCMV). DCs isolated from brains of female mice with LCMV infection were considerably more activated, as shown by increased surface expression of MHC class II and CD86 in female compared to male mice. Exogenous androgen administration to female mice or gonadectomy of male mice resulted in better response to the LMCV, however, neither resulted in the alteration of the DC population in terms of quantity or activation (78). Therefore, it is likely that in this case the immunomodulatory effects of androgens were not directly influencing the DC population.

A study of men with partial androgen deficiency showed that testosterone replacement led to decreased *ex vivo* production of proinflammatory cytokines (79). Another study was performed in men with hypogonadism, also known as testosterone deficiency, where the authors compared the distribution and functional status of peripheral blood (PB) monocytes and DCs (CD16^+^) among other cell types compared to male control subjects. Interestingly, it was found that serum testosterone levels among hypogonadal men were negatively correlated with CpG (oligodeoxynucleotides)-stimulated expression of CD107b by CD16^+^ DCs. These data suggest that low testosterone levels could enhance immune response by increasing circulating (activated) CD16^+^ DCs (80). However, as mentioned previously, these findings represent a descriptive correlation and functional studies are necessary to investigate whether or not testosterone has direct effects on DCs. Here, it is of special importance that different hematopoietic and non-hematopoietic cell populations can express aromatase and can thus convert testosterone to estrogen. Hence, estrogen could also be responsible for observed effects exhibited by increased or decreased testosterone levels (13).

### T Cells

T cells originate in the bone marrow, then migrate to the thymus for maturation and selection, and are subsequently exported to the periphery. They build an essential part of the immune system, coordinating multiple facets of adaptive immunity throughout life. The establishment and maintenance of homeostasis, specific, and memory immune responses depends on T cells. These cells present unique cell surface receptors that are created by randomly assorting V-, J-, C-, and D genes. These receptors recognize foreign particles (antigen) by a highly variable T cell receptor (TCR) expressed at the cell surface, allowing T cells to recognize and respond to diverse antigens derived from pathogens, tumors, and the environment. They also maintain immunological memory and self-tolerance and represent major drivers of many inflammatory and autoimmune diseases. Peripheral T cells comprise different subsets of cells. The thymus is the primary site of T cell differentiation into which T progenitor cells migrate from the bone marrow and undergo TCR rearrangement, giving rise to the two major subtypes of T cells: helper and regulatory (CD4^+^) or cytotoxic (CD8^+^) T cells (81).

The effect of androgens on T cells involves two major processes: thymic size and the differentiation of T helper cells. AR expression was detected in CD3^+^, CD4^+^, and CD8^+^ thymocytes, with the highest expression in cytotoxic T cells (48, 49, 82).

It is well-known that androgen deprivation, due to castration or AR deficiency, causes enlargement of the thymus (83, 84). In this context, specific AR deficiency in T cells (T-ARKO mice) has no effect, while AR deficiency in thymic epithelial cells (TEC-ARKO mice) leads to decreased thymus size. Therefore, AR signaling exerts an indirect but potent effect of in T cell development (please see below) (85). In castrated mice, the administration of androgens completely reversed the thymic hypertrophy and significantly increased the total number of thymic cells expressing AR, nevertheless, it is unclear whether this effect is mediated through T cells (86, 87). Androgen treatment induced a rapid thymic involution suggesting a role for these hormones promoting apoptosis thereby influencing the size and composition of the thymus, as well as inhibiting T cell proliferation (84, 88). Testicular feminization mutation (Tfm) mice (C57BL/6J-ATa), which carry a defective AR gene, also show significant thymus enlargement, but in these mice androgen treatment failed to induce apoptosis in this organ. Notably, the apoptotic response to glucocorticoids was present, thus the apoptosis machinery was not compromised suggesting the requirement of a functional androgen receptor for the induction of androgen-induced apoptosis in the thymus (84). Moreover, the importance of the AR expression in thymic epithelial cells (TECs) as modulators of thymocyte development and its need for normal involutional response to androgens has been demonstrated using chimeric C57 mice, which were Tfm mice engrafted with WT bone marrow cells (89). Consistently, mice with specific deletion of AR in thymic epithelial cells (TEC) had an increase in thymic positive T cell selection, resulting in enlarged thymus and increased T cell numbers. Additionally, AR ablation in TECs enhanced bone marrow transplantation engraftment (85). Altogether, as androgens enhance thymocyte apoptosis, they could represent important mediators for thymocyte selection through direct signaling in TECs, and potentially transmit gender-specific features onto the peripheral T cell repertoire. In one study investigating the sexual dimorphism in central tolerance, was demonstrated the importance of AIRE (autoimmune regulator), which is differentially expressed between the sexes in mice. Results showed that murine female TECs express less AIRE compared to TECs from male mice. The role of androgens was confirmed by orchiectomy, where the lack of male hormones phenocopied female AIRE expression levels. Using an AIRE deficient *in vivo* mouse model, a link between sex biased AIRE expression and increased susceptibility of males to experimental autoimmune thyroiditis (EAT) was established (90). In another autoimmune disease study, a similar sex biased effect of AIRE expression in medullar TECs (mTECs) was observed. Here, the enhanced expression of AIRE by androgens in males was correlated with a protective role in an experimental autoimmune encephalitis (EAE) mouse model (91).

Alterations in circulating levels of gonadal steroids not only affect thymus size, but also affect thymic egression of T cells. In one study carried out in a cohort of healthy vs. hypogonadal men before and after testosterone replacement therapy it was observed that hypogonadism is linked with elevated thymic output of T cells. Consistently, this increase in peripheral T cells was reversed by androgen replacement (87). Furthermore, castration of post-pubertal male mice indicated that T cell numbers in peripheral lymphoid tissues are augmented upon androgen deprivation. In addition, T cells isolated from these castrated mice proliferate more actively in response to TCR- and CD28-mediated co-stimulation as well as to antigen-specific activation compared to the same cells isolated from sham mice (92). Thus, androgens inhibit the number and the receptor repertoire of thymic T cells entering the periphery. Similar findings have been obtained in humans with prostate cancer. ADT resulted in an increase of circulating naïve T cells and of Th1-biased phenotypes. In studies using short-term ADT before prostatectomy an increase in oligoclonal T-cell infiltration into prostate tissue was observed (93). However, a study in prostate cancer patients undergoing androgen deprivation therapy showed a correlation of lower testosterone levels with lower CD4^+^ and CD8^+^ T cell counts at all studied time points (94). The reasons for this discrepancy remain unclear at present but it has to be taken into account that androgen deprivation therapy can also inhibit T cells via off-target effects (please see below).

Androgens not only influence the numbers of peripheral T cells but also affect their responses. Thymocytes and lymphocytes isolated from female mice react more effectively than male cells in mixed lymphocyte reactions (MLR). It was also observed that the production of interleukin 2 (IL-2) was higher in stimulated spleen cells from female mice compared to male or female cells treated with testosterone. In addition, castrated male mice showed increased while androgen-treated female mice exhibited decreased efficacy of antigen presentation assessed by increased lymphocyte proliferation in MLR (95). Altogether, these results confirm the suppressive role of androgens at the level of T cell activation.

T helper (Th) cells polarization arises after T cell receptor (TCR) of CD4^+^ T cells interact with the antigen presented by the major histocompatibility (MHC) complex of professional APC. T helper cells (Th0) mainly differentiate into two functional subtypes, Th1 and Th2. Th1 are pro-inflammatory and characterized by the expression of IFN-γ, IL-2, and TNF-α, while Th2 are anti-inflammatory and express IL-4, IL-5, IL-10, and IL-13 (96). Murine Th cells from males tend to have a more pronounced Th2 cytokine profile, while their female counterparts express more Th1 cytokines. Consistently, androgen treatment enhanced production of IL-10 by murine CD4^+^ T cells, thereby creating a shift toward Th2 responses (97). Additionally, androgens exert an overall inhibitory effect on Th1 differentiation by reducing the phosphorylation of STAT4 mediated by IL-12 (98). Moreover, in an induced mouse model of Grave's disease (an autoimmune disorder that results in hyperthyroidism and is caused by autoreactive T cells killing thyroid cells) (99), mice that were treated with DHT before disease induction had significantly lower IFN-γ and IL-2 production, consistent with an immunosuppressive effect of DHT on CD4^+^ Th1 T cells (100).

Taken together, these findings may explain the lower incidence of autoimmune disease as well as the increased tendency to viral infections in males.

### Treg Cells

Regulatory T-cells (Treg cells) are one of the most versatile immunosuppressive cell population and act as immunological sentinels in different tissues. Lack of Tregs in male mice and men lead to immune tolerance failure and autoimmunity in different organs. Consistently, Treg show continued AR expression after differentiation (50, 101).

Fijak et al. demonstrated that *in vitro* testosterone treatment of naive T cells resulted in an expansion of rat murine Treg cells with immunosuppressive activity. Moreover, in the same study it was observed that substituted testosterone levels in experimental autoimmune orchitis (EAO) in rats significantly increased the number of Treg cells (CD4^+^CD25^+^Foxp3^+^) compared with EAO control animals (102). The same effect was observed *in vivo*, in a systemic lupus erythematosus mouse model, where DHEA administration restored normal levels of Tregs (103). The transcription factor Foxp3 represents a master regulator of Treg function. Interestingly, it was shown that FoxP3 expression can be modulated by testosterone due to direct AR binding to FOXP3 gene regulatory sequences, which could be directly responsible for the increased number of CD4^+^CD25^+^Foxp3^+^ Treg cells upon testosterone treatment (50). Consistently, in a human study it was shown that in medically castrated men, where testosterone levels were reduced, deficiencies in number and function of Tregs were found (104). These results may lead to better understanding and treatment of autoimmune diseases.

A recent *in vivo* murine study assessed whether the function of immunosuppressive Treg cells is responsible for sexual dimorphism in visceral adipose tissue (VAT). VAT represents a hormonally active fat tissue localized around the internal organs. An immunophenotyping screening of VAT tissue showed that males have significantly higher number of Tregs compared to their female counterparts. Next, RNA-seq analysis from male Treg cells isolated from either VAT or spleen tissue showed significant differences in the transcriptional profile involving important regulators of the immune system such as Klrg1, Ccr2, IL-10, or Gata3, between these tissues. Notably, this effect was not observed in female Tregs isolated from the same tissues. Interestingly, this male-specific effect was regulated by androgens through increased numbers of IL-33 producing stromal cells because IL-33 leads to recruitment and expansion of Tregs cells in VAT (105).

### B Cells

B cells form the center of the adaptive humoral immune system and are responsible for the production of antigen-specific immunoglobulins (Ig), commonly known as antibodies. Antibodies are directed against and can clear invasive pathogens (106). Early B cell development occurs in the fetal liver prenatally, before continuing in the bone marrow throughout life.

It is well-described, that regardless of age, females display in general higher numbers of B cells and basal immunoglobulin levels, resulting in greater antibody responses than males. Based on these facts, antibody responses to bacterial infections and viral vaccines are often stronger in females than males, thereby influencing susceptibility of males and females to various malignancies, autoimmune, and infectious diseases (1). Estrogens have been associated with a higher prevalence of autoimmune diseases in females, in which the role of B cells involves different cellular functions, including secretion of autoantibodies, autoantigen presentation, and secretion of inflammatory cytokines (107).

In order to better understand the mechanism of the stronger immune response in females, Furman et al. analyzed the antibody response to a trivalent inactivated seasonal influenza vaccine (TIV) in 37 male and 54 female human subjects. The results showed higher secretion of inflammatory cytokines which could be responsible for enhanced antibody response to TIV in the plasma of women compared to men. Interestingly, a correlation was found between a cluster of genes involved in lipid metabolism, testosterone levels and the response to TIV in men. Thus, those men with higher testosterone levels showed increased expression of these genes accompanied by a poor response to vaccination. This effect was not seen in men with low levels of testosterone or women (108).

Another recent study carried out in mice, showed a sex biased B cell positioning in germinal centers (GCs) representing regions inside the secondary lymphoid organs where B cells can proliferate and mature. The fact, that female B cells are more efficiently positioned within GCs can result in a stronger humoral immune response and also enhance the prevalence to autoimmune diseases displayed in females. The binding of CCL-21 (chemokine ligand 21) to GPR174 receptor expressed on B cells leads to increased male biased migration of B cells toward the periphery of GCs. Consistently, castrated male mice showed a defective GPR174/CCL-21 driven migration, and this effect was rescued upon testosterone replacement. In the same way, female mice supplemented with testosterone mimicked male B cells migration patterns, indicating a sex biased androgen mediated mechanism in B cell immunity (109).

It was reported that the absence of AR expression in B cells, regardless of mouse strain, as well as in castrated WT mice, resulted in an elevated number of B cells in blood and bone marrow (52, 110). Similarly, castrated male mice exhibited higher number of fibroblastic reticular cells expressing BAFF, an essential factor for the survival of B cells. Consistently, the blockade of BAFF receptor by an antibody in male mice phenocopied the enhanced B cell numbers induced by castration. Interestingly, assessment of serum BAFF levels in healthy men showed a correlation of high levels of this cytokine with low levels of testosterone indicating translational relevance of this immunosuppressive mechanism (111).

It is known that AR is expressed in B cell progenitors but not in mature or peripheral B cells, therefore they are sensitive to androgens primarily during development (112). Thus, physiologic levels of androgens regulate in part the production of B lymphocytes, and increased B cell numbers occur in conditions when androgen levels are decreased.

The effect of androgens/AR on B lymphocytes was further confirmed by Altuwajri et al., who observed higher levels of immature B cell development in G-ARKO (global AR knockout) mice comparable to observations in Tfm mice, and also in castrated BALB/c mice. DHT pellets implantation restored the normal B cells levels in bone marrow of castrated mice but not in G-ARKO mice, supporting the hypothesis that androgen-mediated B cell maturation is AR dependent (110). In another study, conditioned medium generated from DHT-treated bone marrow derived cells (BMDCs) resulted in inhibited B cell colony formation. However, this capability was not altered when the conditioned medium was harvested from DHT-treated BMDCs of Tfm mice, proposing an important role for AR in BMDCs mediating the observed differences of B-cell numbers (113).

Furthermore, castration has been shown to significantly increase spleen weight, as well as the total number of peripheral blood B lymphocytes. The increase in circulating B cells was largely due higher numbers of B cell progenitors in the bone marrow with a B220(lo^+^) CD24(hi^+^) phenotype, and this increase was sustained in castrated mice for at least 54 days. After quantifying B cell progenitors in the bone marrow, it was observed that relative numbers of these cells responding to IL-7, including early pro-B cells, late pro-B cells, pre-B cells and immature B cells, were significantly raised. Therefore, androgen deprivation mainly augments numbers of IL-7-responsive B cell progenitors (114).

It is well-described that puberty represents the peak at which sex steroids influence the difference between sexes. However, the impact of sex hormones can begin as early as *in utero*, which could lead to sex disparities in different immune cell populations very early in life. DHT actions *in utero* could influence already peripheral B-cell maturation due to the higher levels of this hormone among boys in the cord blood. In this context, there is one study showing different proportions of immature CD5^+^ B cells between boys and girls already at the age from 3 to 8 years. In one study, testosterone and DHT levels were measured in blood samples obtained at birth and at 8 years of age. Here, a positive correlation between DHT levels at birth and higher proportions of CD5^+^ and immature B cells indicating delayed B cell maturation was found in 8-year-old boys (115).

Together, these findings illustrate the importance of androgen/AR in B cell homeostasis, pointing to the fact that androgens inhibit B lymphopoiesis.

## The Effects of Androgens in Cancer

In cancer the immune system fails to mount an adequate response to combat malignant cells (55), therefore cancer is also the result of failed immune surveillance amongst other causes. There are several studies showing that cancer incidence and mortality is higher in males compared to females, with the exception of few entities including thyroid and gallbladder cancer (2). As we described in the previous section, androgens affect the number, and function of different immune cells (Figure 1). Next, we will review the actions of androgens and AR in prostate cancer (Figure 1). We will focus on this malignancy because in prostate cancer the role of male hormones and its receptors have been the most extensively studied and have the highest clinical relevance. Due to this fact, a substantial number of inhibitors were developed in order to treat prostate cancer. The aim of these therapies is to decrease male hormone levels and AR signaling activation, since this axis is promoting tumor progression. Different androgen deprivation drugs exist and they can be classified in two main classes depending at which level androgen/AR signaling is blocked (116, 117).

**Figure 2:**
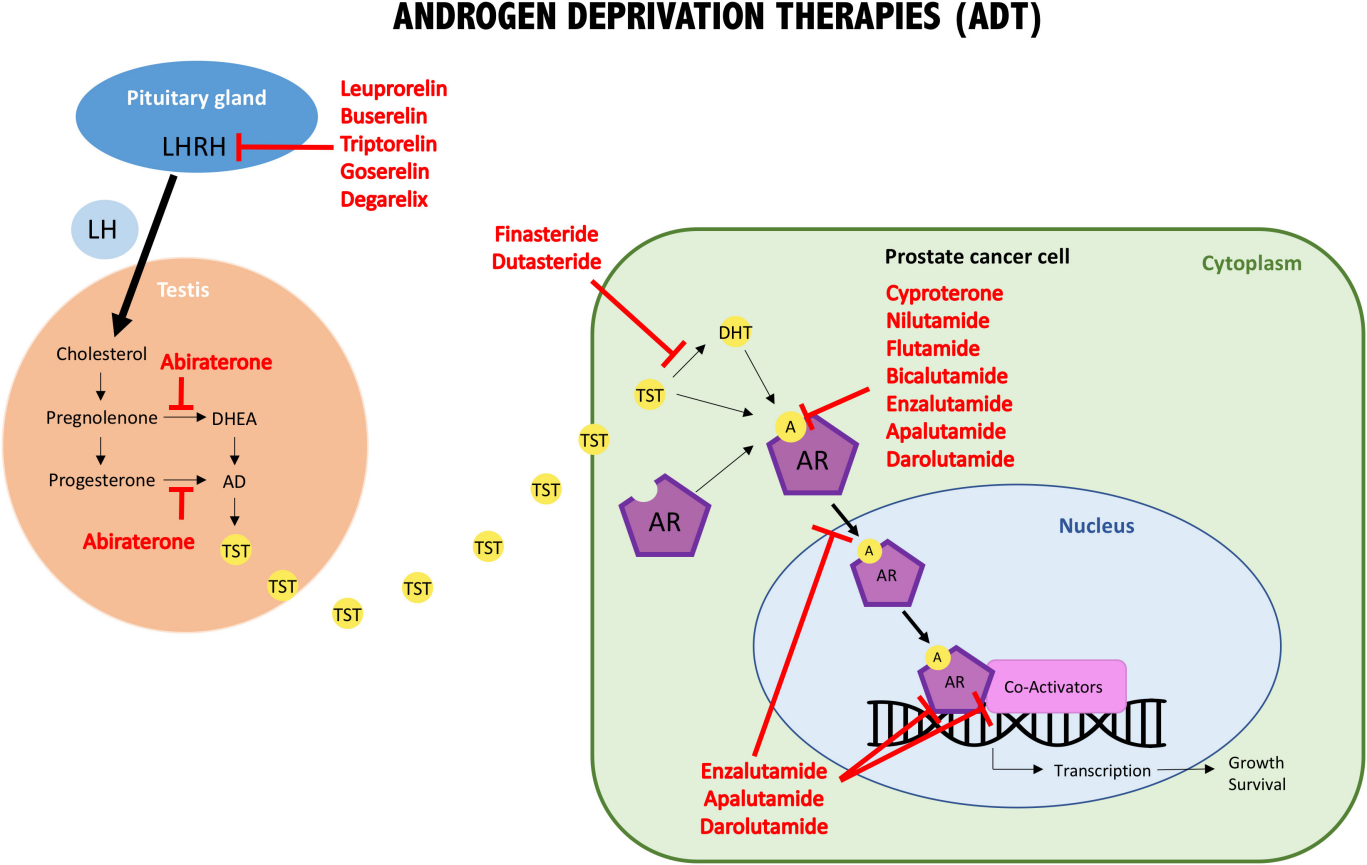
Schematic illustration of androgens/AR interaction, intracellular pathway, and molecular targets of androgen deprivation therapies (ADT). One important group of ADT drugs are the LHRH analogs. They reduce the release of LH, which promotes TST production by the testis. The other important groups are the anti-androgens, either involved in blocking androgens at the synthesis level or involved in interfering with androgen/AR binding (AR blockers). LH, luteinizing hormone; LHRH, LH releasing hormone; DHEA, dehydroepiandrosterone; AD, androstenedione; TST, testosterone; DHT, dihydrotestosterone; A, androgens; AR, androgen receptor.

The first group comprises luteinizing hormone-releasing hormone (LHRH, produced by the pituitary gland) agonists and antagonists, which were amongst the first therapies developed to reduce the amount of testosterone produced by the testicles. GnRH agonists induce an initial massive gonadotropin secretion, which causes the pituitary gland to become desensitized and consequently leading to dramatic suppression of LH. In contrast, GnRH antagonists directly suppress the receptor by competitive inhibition of LH. The LHRH agonist group comprises the following approved drugs: leuprorelin, buserelin, triptorelin, and goserelin. The only LHRH antagonist approved for the treatment of prostate cancer is degarelix [Figure 2; (116)].

The second group are coined anti-androgens because they inhibit androgen synthesis and/or block the binding between androgens and AR. Approved anti-androgens that target androgen synthesis are: abiraterone (which inhibits 17α-hydroxylase), finasteride and dutasteride (which block 5α-reductase action). The first generation of anti-androgens that block the binding of male hormones to their receptor, or inhibit AR nuclear translocation are: bicalutamide, flutamide, nilutamide, and cyproterone (the only steroidal one). A second generation of anti-androgens was synthesized, which have a similar mode of action as the first generation but show improved potency and efficacy. In addition to the above-mentioned mechanisms, these drugs inhibit AR DNA binding and recruitment of co-activators. They include: enzalutamide, apalutamide, and darolutamide. Finally, galeteronel is the only compound with dual androgen antagonist and biosynthesis inhibitor function, but is still pending approval [Figure 2; (116, 118)].

For a more extensive description of the role of androgens/AR in human cancer besides prostate cancer we refer the reader to comprehensive reviews of this field (119).

### Prostate Cancer

The most well-known and -studied androgen/AR-dependent cancer is prostate cancer. With nearly one in every seven men diagnosed during their lifetime, this cancer is the second most frequent in men. Since Huggins and Hodges proved the dependence of prostate cancer on AR pathway by androgen deprivation, it became obvious that this cancer relies on androgen/AR signaling for proliferation and survival [Figure 1; (120)]. This was confirmed by whole genome association analysis (WGA), demonstrating that those genetic loci identified as potential prostate malignancy promoters contain an accumulation of AR- or AR-coactivator binding sites (121). This finding was corroborated by a study of germline mutations, which identified one mutation in the homeobox transcription factor (HOXB13) known to interact with AR, leading to a 20-fold increased risk of inherited prostate cancer (122, 123).

Notably, the link between testosterone and prostate cancer risk is ambiguous at present. For example, some studies revealed a higher risk of prostate cancer among men with low testosterone levels compared to those men with higher levels (124). However, the link between prostate cancer risk and circulating DHT, testosterone or other sex steroids could not be established, even though many population-based studies with this topic have been performed (125).

For example, a meta-analysis published in 2016 found no relationship between testosterone levels in men and their risk of developing prostate cancer, indicating that prostate cancer risk could be unrelated to endogenous testosterone levels (126). Another meta-analysis showed that testosterone therapy did not increase the risk of prostate cancer nor led to its progression in men who have already been diagnosed (127). Furthermore, testosterone replacement therapy also did not increase the levels of prostate specific antigen (PSA), a protein that is elevated in the bloodstream of men with prostate cancer (128).

In some observational studies, more aggressive prostate cancers have even been linked to lower testosterone levels. However, the extrapolation of information from population-based studies is hampered by the fact that many risk factors for prostate cancer, such as obesity, age and associated insulin excess, are correlated with declines in circulating testosterone (125). For this reason, the connection between the serum levels of testosterone and prostate cancer prognosis can differ depending on the clinical settings (129). Altogether, assessment of testosterone levels in circulation have failed to accurately relate prostate cancer incidence or prognosis.

Many pre-clinical and clinical studies have highlighted the importance of AR in prostate cancer. Mouse xenograft models demonstrate that AR^+^ castration-resistant prostate cancer (CRPC) is sensitive to enzalutamide, an AR inhibitor that competitively inhibits androgen binding to the receptor and consequently inhibit AR nuclear translocation and interaction with DNA but AR^−/low^ CRPC is resistant. Consistently, *in vitro* data have shown that genome editing-derived AR^+^ LNCaP cells are sensitive while AR-knockout cells are resistant to enzalutamide (130). Another *in vivo* study has dissected the function of AR in prostate stromal and epithelial cells. To achieve this aim two mouse models were generated: inducible-(ind)ARKO-TRAMP, in which the AR was knocked down in both cell types, and prostate epithelial-specific ARKO TRAMP (pes-ARKO-TRAMP), in which the AR was knocked down only in the prostate epithelium. Findings in both mouse models indicate that the lack of AR leads to less differentiated primary tumors. Interestingly, the results obtained at initial stages in ind-ARKO-TRAMP mice showed less proliferative prostate tumors with smaller size while tumors generated in pes-ARKO-TRAMP mice were proliferating faster and thus larger prostate tumors were present. These data indicated that an early stage of tumor development, the main player involved in primary tumor growth is the prostate stromal AR rather than the epithelial AR. The possible dual roles of androgen action may require reevaluation of ADT regimens, regarding target, and timing in the treatment of prostate cancer patients. These results underline the necessity to develop new selective drugs to specifically target stromal AR in prostate tumors, at least at early stages (131).

Unfortunately, androgen deprivation therapy (ADT) ultimately leads to resistance development in prostate cancer patients. Castration resistant prostate cancer (CRPC) becomes evident after a median of 18–24 months of ADT. In this stage cancer cells are able to proliferate independent of testosterone mostly through androgen-independent AR signaling. Even after ADT, when testosterone is almost not present in the serum of the patients, the AR pathway activation is maintained by different mechanisms, such as upregulation of AR expression, production of androgens outside of the gonads including within the tumor tissue itself, induction of AR mutations leading to ligand independent activation and changes in the coregulator profiles (132).

## The Role of Androgens in Anti-Cancer Immune Therapy

The immune system plays an important role in tumor biology because it can influence essential steps of tumor development like growth, invasion, and metastasis. Tumor cells employ different mechanisms to evade immune elimination, which include: loss of antigenicity, loss of immunogenicity, and orchestration of an immune suppressive microenvironment (133). For this reason, cancer progression occurs in the context of failed immunosurveillance and therefore, strategies designed to harness the natural abilities of the immune system have become very promising approaches to treat cancer.

The principal strategy of cancer immunotherapy is to (re-)boost the immune system against tumor cells in order to allow clearance of malignant cells through mono- or combination treatments. Many different types of cancer immunotherapies exist and are divided in two groups according to their passive or active nature. Passive immunotherapies comprise monoclonal antibodies that target tumor specific antigens. Active immunotherapies represent the larger group including: cytokines/adjuvants, immune checkpoint inhibitors, adoptive CAR T cell transfer, and therapeutic cancer vaccines (134). Due to the outlined overall inhibitory effect of androgens/AR on immune cells it is likely that they also influence the response to anti-cancer immune therapies. In the next section we will overview what is known concerning these hormones and sex-biased differences in the response to immune therapies in mice and humans. Furthermore, preclinical and clinical data of combined ADT and immune therapies are reviewed with a focus on prostate cancer.

### Preclinical Data

Nowadays, immune checkpoint blockade has become one of the most promising cancer immunotherapies. In the preclinical setting, there are no studies yet documenting a direct role of androgens on responses to immune checkpoint inhibitors. However, sex biased response to PD-L1 blockade was observed by Lin et al. In this study, they showed in a preclinical model, in which mice were injected with B16F10 melanoma cells that anti-PD-L1 treatment significantly reduced tumor growth in female compared to male mice. This effect could be partially explained by the inhibited Treg function upon PD-L1 blockade in female mice and enhanced immune response compared to male mice (135). This study suggests that androgens present in males could have a negative impact on the response to anti-PD-L1 treatment—although other underlying mechanisms are also possible.

Type 2 innate lymphoid cells (ILC2) originate from common lymphoid progenitors. They play a crucial role in regulating type 2 inflammation in response to infections with parasites and can promote allergic processes. ILC2 cells are located in different mucosal tissues, like lung, or colon for example, but also in other tissues including liver, fat tissue, and bone marrow among others (136). In these cells, expression of AR has been reported and interestingly the frequency of ILC2 cells in the lungs is sex biased, with higher numbers in females compared to males. Consistently, in castrated mice this cell population in lungs was present in higher numbers compared to male mice. This enhanced presence of ILC2 cells could be one reason for to the enhanced susceptibility of women to develop asthma (137, 138). In the context of cancer, it has been published recently that ILC2 cells can infiltrate human and mouse pancreatic adenocarcinomas and are then designated TILC2 cells. Interestingly, TILC2 cells express the PD-1 receptor. Using a pancreatic mouse model, it was observed that mice treated with anti-PD-1 antibodies exhibited an increased number of TILC2 cells. These cells enhanced tissue-specific tumor immunity by priming CD8^+^ cells and recruiting DCs. Moreover, augmented TILC2 frequencies were associated with longer survival of mice (139). This important study identified activated TILC2 cells as a target of anti-PD-1 immunotherapy and due to their sex biased number in lungs it will be of interest to determine if these cells are involved in mediating sex differences in response to ICI.

Adoptive T cell transfer represents another attractive novel option in cancer treatment. Interestingly, one in *vitro study* indicated an additive effect of ADT and CAR-T cells. Here, T cells were engineered to recognize the aberrantly expressed prostate tumor protein Muc1 and were subsequently able to specifically lyse PC3 cells. Moreover, the combination with the anti-androgen flutamide, was feasible and led to additive anti-tumor effects compared to either therapy alone (140).

Other immune therapies such as therapeutic vaccines have been studied in combination with androgen ablation leading to interesting findings. For instance, in a spontaneous prostate cancer mouse model (TRAMP), a yeast-based vaccine expressing Twist antigen (present in metastatic cells which underwent EMT) was combined with the AR antagonist enzalutamide, resulting in improved survival compared to either monotherapy or untreated control group (141). Another study in a prostate cancer mouse model showed that castration, although not sufficient to prevent invasive and resistant tumor growth, elicited enhanced T cell numbers within the prostate tumors as well as a higher CD8^+^/Foxp3^+^ T cell ratio. Anti-CD25 was used to induce additional Treg depletion, but proved to be insufficient as monotherapy in terms of immunostimulation. For this reason, a second therapy was added which was based on intraprostatic injection of tumor cells expressing an antigen called LIGHT, which is able to recruit and activate T cells to the tumor site, causing rejection of antigenically unrelated tumors. Results showed that combination of castration with both anti-CD25 and LIGHT cell vaccine was more effective in reducing tumor burden and preventing tumor recurrence, compared to castration plus either monotherapy. This improved efficacy was due to immune modulations preventing Treg accumulation and augmentation of effector cells infiltrating the prostate epithelium (142).

Nevertheless, the relationship between androgen deprivation and immunization is not always straightforward. In a mouse study of prostate cancer therapy with ADT and DNA vaccination, *ex vivo* analysis of isolated DCs from the spleens and lymph nodes of castrated and sham-castrated mice showed that simultaneous androgen deprivation increased DCs numbers, but did not improve their costimulatory function for cytotoxic T cells. However, if castration was performed after immunization, androgen ablation was able to increase the immune response elicited by vaccination resulting in increased DC function and T cell cytotoxicity (143).

From a slightly different perspective, a study by Olson et al. showed that prostate cancer cells express higher levels of AR upon androgen deprivation, which in turn improves recognition of tumor cells by AR-specific T cells (144, 145). Therefore, direct targeting of the AR could be a promising immunotherapeutic approach. In line with this hypothesis, immunization of HHDII-DR1 mice, which express human HLA-A2 and HLA-DR1, with a DNA vaccine encoding the androgen receptor, pTVG-AR, augmented HLA-A2-restricted immune responses. This led to lysis of syngeneic prostate tumor cells, resulting in a reduction of tumor burden concomitantly with an improved overall survival of tumor-bearing mice (146).

Even though most studies focusing on androgen ablation in combination with cancer immunotherapy have been performed in prostate cancer for obvious reasons, a study by Hsueh et al., found that androgen blockade enhances the response to a melanoma vaccine in a syngeneic murine model. Here, the combination of flutamide treatment followed by irradiated cell vaccine, prior to melanoma inoculation, resulted in better survival rate compared to either flutamide or vaccine alone, as well as to the untreated group (147).

Altogether, preclinical data suggest that androgen deprivation therapy (surgical and medical) could potentially be used in combination with different kinds of immunotherapies. However, it is important to note that there is a caveat regarding certain medical ADT therapies. In a study published by Pu et al., it was observed that orchidectomy in combination with CpG vaccine was beneficial in terms of survival and immune response in a murine model of prostate cancer. However, some AR antagonists (flutamide and enzalutamide) showed unexpected immunosuppressive effects when given in combination with the same vaccine. This immunosuppressive effect was likely due to an elusive off-target effect on T cells, leading to impaired activation. This led to ineffective immunization when given simultaneously but not when applied before ADT. Notably, the use of alternative ADT therapies such as androgen biosynthesis inhibitors in combination with CpG vaccine showed success in synergistic inhibitory effects on cancer tumor growth in mice (148). These findings illustrate the importance of meticulous preclinical research in order to optimize combination partners and timing of combined immuno- and ADT, which should inform the design of rational clinical trials.

### Clinical Data

Immune checkpoint inhibitors (ICIs) lead to promising outcomes in some but not all cancer entities. Because of the importance of this treatment modality the following section will focus on what is known concerning the influence of male sex (and therefore possibly androgens) on the therapeutic response to ICIs in humans.

ICIs mostly withdraw inhibitory signals of T-cell activation, thereby tumor-reactive T cells are able to surpass negative regulatory mechanisms and exert a more potent antitumor immune response (149). Currently, monoclonal antibodies targeting T-lymphocyte-associated antigen-4 (CTLA-4), programmed-death 1 and programmed death-ligand 1 (PD-1 and PD-L1) represent key ICIs. They have been already approved for certain entities including lung-, bladder-, kidney-, skin-, and head-and-neck cancer amongst others (150, 151). Today, many phase I-III clinical trials are being carried out worldwide to evaluate the efficacy of multiple ICIs as mono- or combination therapy for many different cancers (150).

Despite encouraging findings, low response rates were observed in some tumors. For example, treatment with ICIs in melanoma and non-small cell lung cancer has relatively high efficacy while in other entities such as pancreatic cancer, breast cancer and many sarcoma entities, response rates remain low. Moreover, only a relatively small part of cancer patients experience long-term benefit from ICI treatment and a significant number of patients experiences immune-related adverse events during therapy. This is not too surprising because inhibition of immune checkpoints can cause autoimmune responses to healthy tissues (152, 153). Thus, there is a need to develop predictive biomarkers in order to differentiate responding and non-responding patients, to reduce adverse effects and possibly anticipate the requirement of combination therapy for patients unlikely to respond to ICIs (154).

As described in the first section of this review, it is known that sex is a variable affecting both innate and adaptive immune responses (1). Nevertheless, it is alarming that <10% of cancer immune therapy-related data are analyzed taking into account the sex of the animal or human subjects (155). This is even more concerning because the available meta-analyses of large ICI trials in different entities suggest that ICIs could show different efficacy according to the sex of cancer patients, pointing to better results in males than females (151). However, the sex bias in response to anti-cancer immune therapies is an ongoing matter of debate and could not yet be resolved.

One important meta-analysis in this context has been carried out by Conforti and colleagues. The authors assessed the difference in ICI efficacy between men and women from 20 randomized controlled trials of ICIs (ipilimumab, tremelimumab, nivolumab, or pembrolizumab) including more than 11.000 patients showing overall survival according to the sex of the patients. The study contained patients with different advanced or metastatic cancers (67% men and 33% women) and the most common cancer entities were melanoma (32%) and NSCLC (31%). This analysis revealed a higher reduction in the risk of death in males compared to females upon treatment with the different ICIs. Most importantly, it was reported that overall survival was improved by these therapies for all patients, but that the magnitude of benefit is sex-dependent (156).

However, in a different meta-analysis of 23 randomized clinical trials, in which ICIs were used, 7 more additional clinical trials were included compared to the meta-analysis described before (156). In this study more than 13.000 patients were analyzed of whom 68% were men and 32% women. An overall survival benefit upon treatment with ICIs alone was found for both men and women with advanced solid malignant neoplasm (48% NSCLC, 17% melanoma, 9% renal cell carcinoma, 9% SCLC, 4% urothelial, gastric, head, and neck squamous carcinoma and mesothelioma). In this analysis, no statistically significant differences between the sexes were observed (151).

After showing that men obtained larger benefit than woman from therapy with anti-CTLA-4 or anti-PD-1 agents, Conforti et al. performed a second study where the authors investigate whether the combination of chemotherapy and anti-PD-1 or anti-PD-L1 could be more effective in woman compared to men. The meta-analyses were conducted with data from 11 randomized controlled trials comparing progression free survival (PFS) and overall survival (OS) in patients who received combination of ICI therapy with chemotherapy with those who were treated with ICIs or chemotherapy alone. In this study, the results concluded that women had better responses to the combination of ICI and chemotherapy compared with men, while men responded better to either chemotherapy or ICI therapy alone compared with women (157). A recent paper published in by Ye et al. addresses the issues regarding the conflicting results generated by meta-analyses regarding sex differences in response to ICB. They point to the fact that due to the substantial heterogeneity of the clinical trials included, especially considering the control arms, a meta-analysis approach was not the proper analysis to be performed on these data sets as a whole, as effects could have been masked or diluted. As a result, they decided to take a different approach and used ICB treatment data sets with molecular profiling for individual patients. In this way, they were able to observe divergent patterns in overall survival (OS) between males and females through different cancer entities in response to ICB. Regarding anti-PD-1/PD-L1 therapy male patients with colorectal cancer or glioblastoma multiforme showed increased survival, while female patients with esophagogastric cancer (ESCA) or NSCLC tended to have better OS. These gender differences were attributed to a number of factors including tumor mutation burden, neoantigen load, and mutation rates, which themselves showed gender disparity. More importantly, they demonstrated that it is of great importance to separate each cancer entity instead of pooling them together, as this could be one of the biggest barriers in properly analyzing the influence of sex in ICB therapy response (158).

Altogether, the data concerning a sex-bias in the response to current ICI treatments are ambiguous at present. Reasons for this include multiple statistical caveats with meta-analyses including publication bias and inhomogeneity in the statistical design of clinical trials. Given the documented impact of sex on immune responses, it is hard to understand why in most ICI clinical trials a substantially larger fraction of males was included which could lead to bias in the results. Trials with equal numbers of males and females stratified for sex and/or separate trials in the different sexes are warranted especially in the context of anti-cancer immune therapies.

Altogether, the combination of ADT and immune therapy could open interesting therapeutic options especially in patients with androgen/AR-dependent cancers. Therefore, we now summarize the available clinical information about different immune therapies in prostate cancer with a focus on combination with ADT.

### Combination of ICI and ADT in Prostate Cancer Patients

To date, many efforts have been made to integrate immunotherapy in the course of treatment of advanced prostate cancer. However, most immunotherapeutical approaches did not fulfill the high expectations. A rather “cold” tumor microenvironment and a low tumor mutation burden have been identified as potential causes. As mentioned above, ADT routinely used for advanced disease was found to influence the immune system in both, positive and negative ways (159).

So far, most trials have been carried out in androgen independent PCa characterized by disease progression despite testosterone values in the castration level due to ADT or orchiectomy. In fact, the only immunotherapy approved to date is Sipuleucel-T for asymptomatic or mildly symptomatic castration resistant prostate cancer (CRPC). Sipuleucel-T is an autologous cellular immunotherapy for which DCs are incubated *ex vivo* with a fusion protein consisting of prostate specific acid phosphatase (PAP) and granulocyte-macrophage colony-stimulating factor (GM-CSF) (160). In a phase 3 clinical trial (IMPACT; NCT00065442) with mCRPC patients, it was shown that in patients treated with Sipuleucel-T, the risk of death was significantly diminished and median overall survival (OS) was increased by 4.1 months vs. placebo-treated patients. However, due to company policy Sipuleucel T is only available in the USA and Canada.

In addition, different vaccination strategies were developed for prostate cancer. ProstVac VF (PRO; PSA-TRICOM), is a heterologous prime-boost regimen of two different recombinant pox-virus vectors that comprises a prime and multiple boosts with attenuated strains of vaccinia and fowlpox viruses, respectively. Both recombinant viruses encode human PSA and T-cell co-stimulatory proteins CD54, CD58, and CD80 (TRICOM). Remarkably, in a placebo-controlled phase 2 clinical trial of men with minimally symptomatic and chemotherapy-naive mCRPC, PROSTVAC was linked to a 44% decrease of death (161). In contrast, a double-blind, randomized phase 3 clinical trial evaluating 3 treatment groups (1) PRO+ Placebo, (2) PRO+GM-CSF, or (3) Placebo + Placebo showed no survival advantage with a median OS of 34.3, 33.3, and 34.2 months, respectively (162).

The first ICI examined in mCRPC was the CTLA-4 inhibitor ipilimumab (ipi). In a multicenter, randomized, placebo-controlled, double-blind, phase 3 trial ipi was evaluated in men with at least one bone metastasis originated from CRPC who had progressed after docetaxel therapy. Patients received first bone-directed radiotherapy and then were treated with either ipi 10 mg/kg or placebo every 3 weeks for up to four doses. Median overall survival was 11.2 and 10.0 months after ipi or placebo, respectively, with a trend for improval, but no significant advantage for patients treated with the immunotherapy (HR 0.85; *p* = 0.053) (163). In a second multicenter, double-blind, phase III trial, ipi was compared to placebo in chemotherapy-naïve mCRPC patients. Ipi 10 mg/kg or placebo were administered every 3 weeks for up to four doses followed by maintenance therapy in non-progressing patients every 3 months. Again, the study failed its primary endpoint with no survival advantage for Ipi (164).

Similarly, results of PD-1 inhibitors in unselected PCa patients have been rather disappointing. Thus, PD-1 inhibitor pembrolizumab (pembro) monotherapy in PD-L1 positive mCRPC achieved CR, PR and stable disease in only 2, 4, and 17% of the patients, respectively, while 58% of the men were primarily progressive (165). Results for PD-L1 negative patients were even worse with no CR, 3% PR and 63% progressive disease. Interestingly, an upregulation of PD-L1 was observed in patients developing resistance to AR targeting agent (ARTA) enzalutamide (enza) (166). Enza effectively inhibits androgen binding to its receptor, AR nuclear translocation and subsequent interaction with DNA. It is widely used for the treatment of advanced prostate cancer with approvals for the treatment of metastatic and non-metastatic CRPC as well as hormone sensitive PCa (Dez 2019; FDA only). As described above, androgen deprivation has been associated with T-cell tumor infiltration and activation as well as increased T-cell responses in preclinical models (93, 98). Consequently, the addition of pembro was evaluated in a phase 2 clinical trial in patients progressing on enza. Remarkably, a PSA-response >50% and radiological responses were observed in 18 and 25% of the patients [Figure 1; (167, 168)]. An expansion cohort with 30 additional men presented at last year's ESMO confirmed these results with PSA- and radiological responses in 20 and 22% of the patients, respectively (169). Based on these results, phase 3 clinical trials evaluating the combination of Pembro and Enza have been initiated in hormone sensitive and castration resistant advanced prostate cancer.

In addition, different combinational treatment strategies, e.g., with different immunotherapies or IO and chemotherapy are currently under investigation in mCRPC and showed first promising results (Table 1).

**Table 1:** Ongoing clinical trials combining ADT and immunotherapies in prostate cancer.

**Indication**	**Drug**	**Phase**	**Study**	**No. of patients; primary endpoint**
mCRPC	Enzalutamide + Atezolizumab vs. Enzalutamide	III	NCT03016312, Imbassador 250	*n* = 771; OS
mCRPC	Enzalutamide + Pembrolizumab	II	NCT02312557	*n* = 58; PSA response
mCRPC	Enzalutamide + Pembrolizumab vs. Pembrolizumab	II	NCT02787005, Keynote 199	*n* = 370; ORR
mCRPC	Enzalutamide + PROSTVAC-F/V-TRICOM vs. Enzalutamide	II	NCT01867333	*n* = 57; TTP
mCRPC	Abiraterone Acetate + Prednisone + Ipilimumab	I/II	NCT01688492	*n* = 57; PFS, safety
mCRPC	Effect of fecal transplantation from responders to Pembrolizumab/Enzalutamide to non-responders	II	NCT04116775	*n* = 32; PSA response
mCRPC	4 arms: Pembrolizumab + Olaparib; + Docetaxel + Prednisone; + Enzalutamide; + Abiraterone + Prednisone	Ib/II	NCT02861573, Keynote 365	*n* = 400; PSA response, safety, ORR
mCRPC	Enzalutamide + Pembrolizumab vs. Enzalutamide + Placebo	III	NCT03834493, Keynote 641	*n* = 1,200; OS, PFS
mCRPC	Nivolumab + Bipolar Androgen Therapy (supraphysiological testosterone therapy)	II	NCT03554317, COMBAT-CRPC	*n* = 44; PSA response
mCRPC	3 arms: Nivolumab + Rucaparib; + Docetaxel + Prednisone; + Enzalutamide	II	NCT03338790, CheckMate 9KD	*n* = 330; ORR, PSA Response
mCRPC	Abiraterone + Prednisone + Apalutamide vs. Abiraterone + Prednisone + Apalutamide + Ipilimumab	II	NCT02703623	*n* = 198; OS, safety, AR response marker, PSA, CTCs
mCRPC	many arms, different solid tumors: AZD4635 + Durvalumab vs. Durvalumab	I	NCT02740985	295, incidence of DLT in solid tumors
mCRPC	Avelumab + Abiraterone or Enzalutamide	II	NCT03770455	*n* = 13; PSA response
mCRPC	Avelumab + Bempegaldesleukin + Enzalutamide	Ib/II	NCT04052204	*n* = 170; DLT, PSA response
mHSPC	Nivolumab + Degarelix vs. Nivolumab + Degarelix + BMS-986253	Ib/II	NCT03689699, MAGIC-8	*n* = 60; PSA response, safety
mHSPC	ADT + Docetaxel vs. ADT + Docetaxel + Nivolumab vs. ADT + Ipilimumab/Docetaxel + Nivolumab	II/III	NCT03879122, PROSTRATEGY	*n* = 135; OS
CSPC	Ipilimumab + GnRH Analog	II	NCT01377389	*n* = 30; progression after 6 months
CSPC	Enzalutamide + PROSTVAC-F/V-TRICOM vs. Enzalutamide	II	NCT01875250	*n* = 38; tumor growth
CSPC	Degarelix + Ipilimumab	II	NCT02020070	*n* = 16; PSA response
Oligometastatic PC	Abiraterone Acetate + Prednisone + leuprolide acetate + Pembrolizumab + SBRT+/– SD 1-01	II	NCT03007732	*n* = 42; PSA response
Oligometastatic PC, neoadjuvant	Degarelix + Pembrolizumab + cryosurgery	II	NCT02489357	*n* = 12; PSA response, safety
Localized PC, neoadjuvant	Degarelix + Cyclophosphamid + GVAX vs. Degarelix	I/II	NCT01696877	*n* = 29; CD8+ T-cell infiltration, adverse events
Localized PC, neoadjuvant	Enzalutamide + Pembrolizumab	II	NCT03753243	*n* = 32; PCR
Localized PC, neoadjuvant	Atezolizumab vs. Atezolizumab + Enzalutamide	II	NCT03821246	*n* = 51; change in CD3^+^ T-cells

*The table was adapted from Ozdemir and Dotto (6) and Taghizadeh et al. (170). The ClinicalTrials.gov database registry was searched for the terms “prostate cancer” and several CTLA-1 and PD-1, PD-L1 inhibitors. Not yet recruiting trials are not listed. PC: prostate cancer, mCRPC, metastatic castration resistant prostate cancer; mHSPC, metastatic hormone sensitive prostate cancer; CSPC, castration sensitive prostate cancer; ADT, androgen deprivation therapy; AR, androgen receptor; A2AR, adenosine A2A receptor; SBRT, stereotactic body irradiation therapy; OS, overall survival; PSA, prostate specific antigen; ORR, overall response rate; TTP, time to progression; DLT, dose limiting toxicities; PCR, pathological complete response*.

## Concluding Remarks

In summary, the androgen/AR axis plays a crucial role in both reproductive and non-reproductive tissues. AR signaling has been shown to directly and indirectly affect many immune cells types from innate and adaptive immunity. Overall, the effect of androgens is largely immunosuppressive, in terms of cell numbers and activation state. Furthermore, androgens/AR have been associated to poor prognosis in a plethora of cancer entities. However, deprivation of androgen signaling, has not always led to convincing beneficial effects in patients except for prostate cancer, therefore, future studies are warranted to determine specific mechanisms taking place and identify better treatment strategies. Additionally, despite impressive advances in the field of cancer immuno-oncology, the effect of androgens in anticancer immunity is yet to be determined. More importantly, there is lack of knowledge regarding the effects of androgens in emerging therapies. Clinical studies should include the possible effects of sex in the trial design.

## Author Contributions

IB-B, MV-D, GA, MJ, and SL wrote the manuscript. IB-B and MV-D conceived and edited the figure. All the authors approved the submission of the manuscript.

## Conflict of Interest

GA declares the following conflicts of interest: Consulting or Advisory Role: Roche, BMS, Astellas, Sanofi, Janssen, MSD, Merck Serono, Pfizer. Honoraria/Travel Support/Speaker's Bureau: Roche, BMS, Astellas, Sanofi, Ipsen, EISAI, Pierre Fabre, MSD, Astra Zeneca, Janssen. Research Funding (within clinical trials sponsored by the pharmaceutical industry): Roche, BMS, MSD, Astra Zeneca, Sanofi, Incyte. The remaining authors declare that the research was conducted in the absence of any commercial or financial relationships that could be construed as a potential conflict of interest.
